# The Fairy Tale Model: Secure Facility Therapist Perceptions

**DOI:** 10.1007/s40653-018-0203-2

**Published:** 2018-01-19

**Authors:** Ian Barron, David Mitchell

**Affiliations:** 1 School of Education and Social Work, University of Dundee, Nethergate, Dundee, DD1 4HN UK; 2 Rossie Young People’s Trust, Montrose, Angus UK

**Keywords:** Incarcerated youth, Trauma recovery, Therapy, Evaluative research

## Abstract

The current exploratory qualitative study sought to investigate novice therapist experience of implementing a phased trauma recovery approach, the Fairy Tale Model (FTM), in secure accommodation in Scotland. Participants were ten therapists trained and supervised in FTM over a 6 month period. Therapists delivered FTM to 37 youth. Individual interviews with therapists were based on the objectives of FTM, and explored the benefits, challenges and facilitating factors for both youth and therapists. Perceived benefits for therapists included increases in trauma-informed knowledge, skills, and confidence. Youth were perceived by therapists, to be less emotionally dysregulated and more motivated, hopeful, and communicative. Challenges for therapists involved the complexity of youth difficulties, competing work demands, difficulties unlearning established approaches, and short duration placements. Prioritizing therapy, intensive sessions, and frequent communication with care staff were seen as facilitating factors. Recommendations are made for FTM delivery and more robust mixed methods evaluative research including therapist, youth and other stakeholder perspectives.

The current study evaluates the perceptions of the first individualized trauma-informed phase approach to be introduced into secure youth facilities in Scotland. Prior Scottish studies had indicated high levels of traumatic exposure and resultant trauma symptomology in youths in secure accommodation (Barron and Mitchell 2017a, b). In addition, a group-based cognitive behavioral therapy intervention had been implemented but evidenced the need for individual therapy for some adolescents (Barron et al. 2016). As a consequence, it was decided to implement an individualized trauma-informed phase approach, the standard of care for treating traumatization (Foa et al. 2009). The Fairy Tale Model (FTM) (Greenwald 2009) was adopted because of its development with youth in residential facilities. The approach builds on the common factors research of effective therapies. FTM and its components are empirically based and listed by the California Evidence-Based Clearing House (Greenwald 2013). In selecting FTM, the current authors considered this approach to be more attuned to the needs of youth in secure facilities, compared to other phased approaches developed with community populations, e.g. Cognitive Behavioral Therapy or Eye Movement Desensitization Reprocessing (Shapiro 2001). Common factors research sought to identify features that are associated with effective therapies. FTM incorporates and promotes these factors (Greenwald 2013). Firstly, FTM is experienced positively by clients and therapists, and aims to facilitate the building of therapeutic alliance (Duncan et al. 2010). Through the use of scripted case formulation and treatment contracting, FTM seeks to develop shared perspectives and planning (Messer and Wampold 2002). Active client agency (Bohart and Tallman 2010) is enabled by the identification of client strengths and facilitating choices, and regular feedback (Lambert 2010) is embedded in the therapeutic process prior to treatment, at mid-therapy check-ins, and at the end of therapy. Finally, FTM’s focus on client strengths, containment of emotion, and belief in a client’s ability to problem solve and process their trauma, supports the common factors of empathy, warmth, and positive regard (Norcross 2010). With trauma, however, common factors have found to be necessary, but not sufficient for effective resolution. Processing the trauma, for example, is a necessary component (Ecker et al. 2012). In addition to common factors research, Greenwald (2013) reports there is considerable evidence underpinning the components of FTM including motivational interviewing, cognitive behavioral training, attachment, trauma resolution, and relapse prevention. Although different protocols exist for motivational interviewing, there is sufficient consistency of approach to assert effectiveness, at least in the short term (Strait et al. 2012). Numerous studies have affirmed the efficacy of cognitive-behavioral skills training for a wide range of mental health problems (Nathan 2007), and there is extensive literature affirming the centrality of working with attachment in therapy (Orlans and Levy 2014). Progressive Counting, typically used in the trauma resolution phase of FTM, has a growing empirical base (PC: Greenwald et al. 2013, 2015; Barron and Tracey 2017). Finally, early studies into alcohol and substance misuse, and more recently studies related to other mental health concerns, highlight the importance of planning for relapse prevention (Marlatt and Donovan 2005). FTM as a whole package has been evaluated and found to be a supportive experience that leads to positive outcomes for youth. Within the residential setting, symptoms of posttraumatic stress and incidents of violence have halved and discharge rates from residential to community care doubled (Farkas et al. 2010; Greenwald et al. 2012). Mental health and behavioral gains for abused youth have maintained at 3 months and 6 month follow-up (Farkas et al. 2010; Greenwald 2003). High treatment retention rates have been achieved and treatment times have shortened (Becker et al. 2011; Greenwald et al. 2012). To date, there has only been one FTM study in a secure facility in Scotland. Perceptions of a therapist and the first three youth to successfully complete FTM, incorporating Progressive Counting, were assessed using in-depth interviews. Progressive Counting is a novel approach to brief exposure where the worst part of the trauma image is sandwiched between a positive past and positive future images, while the therapist incrementally increases exposure through counting out loud (Greenwald 2013). Therapist and youth reported gains for youth in all treatment objectives including increased motivation, capacity to assess risk, and the ability to consider positive alternatives. Youth also reported reduced stress levels (Barron and Tracey 2017). There have only been two other qualitative FTM studies, both conducted in the US. Greenwald and colleagues (2003) explored workers in training perceptions of FTM and found training and supervision improved therapist sense of competence and work satisfaction. Similarly, in a later study, Greenwald et al., (2008) discovered worker reactivity reduced and improvements were made in attitude, and worker behavior towards clients. In short, the small cluster of qualitative studies suggests a positive experience and tangible outcomes of youth and workers. Qualitative research is not uncommon in the evaluation of therapeutic experience. A wide range of therapies, including trauma recovery programs, have been evaluated using a variety of methods such as questionnaires (Bussey 2008), interviews (Alisic 2012), and focus groups (Barron and Abdallah 2012). The focus is typically on therapist and/or client perceptions of therapeutic experience and outcomes. There are, however, a number of criticisms of qualitative approaches. For example, it has been suggested that there is uncertainty as to whether participants can always access their own beliefs during qualitative methods (Smith 2015). Researchers have raised questions as to whether participants are accurately describing their lived experience of therapy or if their sense of experience is being constructed in the giving of responses (Hammersley 2005). Some argue that qualitative approaches remain at exploratory and speculative levels (Robson and McCartan 2016). On the other hand, Barron and Topping (2011) caution against implementing outcome evaluations too early as this can undermine promising programs. New initiatives in new contexts, for example, may be best investigated by qualitative approaches to explore potential program outcomes, identify adaptations for future delivery, and the need for more robust evaluative research.

## The Current Study

The current exploratory qualitative study evaluated FTM in the novel setting of secure youth facilities in Scotland. Building on previous studies of FTM in residential settings, and the experiences of youth reported in a cluster case study in a secure facility (Barron and Tracey 2017), the current study explored novice therapist perception of the process and outcomes of FTM in order to inform future secure facility delivery and evaluation. Qualitative semi-structured interviews explored therapist perceptions of the benefits, challenges, and facilitating factors of implementing a trauma-informed phase model into a context that had traditionally used a behavior risk paradigm (Barron and Mitchell 2017a, b). Analysis was quasi-qualitative and sought to quantify and rank order codes of meaning as well as identify themes for the development of practice and future research.

## Methods

### Research Design

The current study was qualitative in nature. Novice therapists, those new to delivering a brief exposure therapy with limited experience, participated in individual interviews and were asked open-ended questions based on FTM objectives. Quasi-qualitative analysis was thematic where statements and codes were identified, counted and rank ordered. Ethical approval for the study was obtained from the University of Dundee University Research Ethics Committee (UREC). Participants provided informed consent. Parents/guardians gave informed consent for minors involved in the study. Minors gave informed assent. Participants were informed they could drop out at any time.

### Participants

Ten therapists from 4 secure facilities were trained over 4 days in the Fairy Tale Model by Dr Greenwald, developer of FTM. Three facilities accommodated 18 youth each, and 1 facility accommodated 24 youth. Youth, in the facilities, were from all over Scotland and recently some were placed from England. Therapists had worked in secure facilities for 2 to 6 years, mostly utilizing behavior change programs, with 4 years’ experience on average. Nine therapists were female and 1 male. Over a 6 month period, therapists saw 37 youth, ranging from 1 to 10, and averaging 3.7 cases. Youth were all Caucasian, aged 12 to 17 years of age and had been placed in a secure facility, on average for 3 months, because of the risks they posed to themselves and others though violence, self-harm, substance misuse, theft, and vandalism. The majority of youth were male. Youth received FTM in place of behavior programs and emotional awareness/regulation training.

### Intervention

The Fairy Tale Model of trauma recovery includes: assessment of the youth strengths and resources, trauma and loss history, life situation, and presenting problems; identification and enhancement of the youth goals and motivation; trauma-informed case formulation and treatment contracting; stabilization (including case management, parent/staff training, problem-solving, and strategic avoidance of high risk situations); identification and enhancement of coping and affect tolerance skills; resolution of trauma and loss memories; consolidation of gains; and anticipation of future challenges (14 sessions in total). Although FTM may include systemic interventions, this project featured individual psychotherapy. Therapists received 11 supervision sessions over a 6 month period involving 7 half days via video conferencing and 4 full days face to face. Supervision involved expert analysis and feedback of practitioners’ videoed sessions with youth. Practitioners were requested to video all therapy sessions and bring the videos to supervision for learning and fidelity purposes.

### Procedure

This study utilized a semi-structured interview with therapists based on questions developed from FTM objectives. Open ended questions focused on the perceived benefits and challenges for youth and their community, practitioners, secure care facilities, care staff, parents, and agencies as well as what facilitated and hindered FTM delivery. The interview was piloted with a practitioner not involved in the study. This resulted in questions being numbered to aid clarity of recording. All interviews were conducted by the principal researcher. Interviews were digitally recorded and transcribed verbatim by an independent company. Interviews were designed to last around 45–50 min. Accuracy of transcription was checked by the principal researcher.

### Analysis

Analyses of interview transcriptions was conducted by the principal researcher, a reader in trauma studies. A novel quasi-qualitative approach involving a six-step systematic thematic analysis was used (Braun and Clarke 2006). The term quasi-qualitative refers to the counting and rank ordering of codes, and statements. The procedure for analysis is as follows: Familiarization involved re-reading the data and noting initial ideas for patterns of meaning and initial codes were systematically generated from the data with statements of meaning collated under each code. Where possible, codes were named using participants words rather than from theory. Codes were collated with the set of statements into identified themes, and codes and statements were counted, and rank ordered. Initial codes and themes were reviewed, and checked against participant statements, and finally, themes were named, and report writing enabled a further level of analysis with the identification of exemplar statements for codes and themes. The name of the codes is reported along with the number of statements for each code. The number of codes per theme is totaled. Cross-question analysis was conducted on the benefits, barriers and facilitating factors to effective practice. Because of the small sample size of secure facilities and therapists, no identifiers were used for individual contributions. Inter-rater reliability involved a post graduate research assistant independently reviewing the statements, codes and themes.

## Results

Inter-rater analysis led to eight themes identified that were sufficiently different to warrant discussion between the principal researcher and the research assistant. Changes involved reducing the wordiness of themes to get to a single main statement. Table 1 summarizes the identified themes.

**Table 1:** Therapist identified themes for adolescents and therapists

Adolescents
- Transformational and positive experience leading to enhanced sense of possibility for change
- Easy application leading to positive adolescent change
- Builds onto previous skills, and materials clear to use
- Gaining knowledge that trauma is treatable
- Discovering strategies to deal with past experience and for positive change
- For most, opened up new ways of thinking and increased awareness of being able to make choices
- Care staff noticed visible changes in attitude and behavior
Therapists
- Increase in understanding, and awareness of trauma exposure and recovery
- Overall increase in assessment accuracy and capacity
- Delivery of therapy more efficient
- Lack of experience in trauma report writing has been addressed for some
- Greater confidence in speaking to others about trauma, but a need for training in involving parents and care workers
- Mixed responses revealed some staff are already trauma-aware
- Trauma now reported in transition meetings
- Lack of skills, time, and resources are a challenge
- Need for flexibility and support to facilitate delivery of FTM

### Benefits Perceived for Youth

Table 2 compares therapist perceptions of the benefits and barriers for youth and therapists, and facilitation factors for implementation. Benefits for youth included fourteen codes were from 41 statements. Codes were: the structure of FTM (*n* = 7); motivating activities: (*n* = 6); planning and working towards future goals (*n* = 6); reduced trauma symptoms (*n* = 5); useful explanations for trauma and resultant behavior, e.g. “sore spot” trigger (*n* = 4); can see how to overcome difficulties (*n* = 4); in charge of their own therapy (*n* = 2); thinking things through (*n* = 1); able to play out as an imagined movie when not able to talk (*n* = 1); an opportunity to see a better ending to a difficult situation (*n* = 1); new skills to get to the good ending (*n* = 1); learned coping skills for risks and triggers (*n* = 1); working on what’s important to them (*n* = 1); and moved on and done well since (n = 1). The theme is: *Transformational and positive experience leading to enhanced sense of possibility for change*.

**Table 2:** Therapist perception: rank order of benefits and barriers to youth and therapists

Youth benefits	Therapist benefits	Barriers	Facilitating factors
FTM structured (*n* = 7)	Structured script (*n* = 12)	Self-directed learning (n = 4)	Capacity for longer sessions (*n* = 5)
Motivating activities (*n* = 6)	Understand trauma (*n* = 11)	Complexity of youth needs (n = 2)	Information to care workers (*n* = 3)
Future goals (*n* = 6)	Confidence (*n* = 8)	Emotional immaturity (n = 2)	Choice of therapist for youth (*n* = 2)
Reduced symptoms (*n* = 5)	Reduced planning time (*n* = 8)	Paradigm clash (n = 2)	Less cases (*n* = 2)
Normalising reaction (*n* = 4)	Addressed trauma (*n* = 7)	Conflicting discipline lens (n = 2)	Intensive sessions (n = *2*)
Overcome difficulties (*n* = 2)	Sought more training (*n* = 5)	Other work demands (n = 2)	
In-charge own therapy (n = 2)	Structured pack (*n* = 3)	Limited care support (n = 2)	
	Treatment plan (*n* = 3)	Staff and role changes (n = 2)	
	Consolidated learning (*n* = 2)	Under-resourced therapists (n = 2)	
	Youth led process (*n* = 2)		

Most therapists emphasized how FTM helped youth see how they could overcome their difficulties through identifying their own treatment goals, coping skills, and avoiding high risk situations.

The youths really got a lot out of it. In particular, working towards their future goals and seeing that you’d overcome the difficulties they’d had, and still being able to achieve things, that’s why it’s particularly effective for them … The structure to the work. It’s good to give them hope for the future and a clear plan towards that. It’s good that it gives some direct coping skills, like avoiding high risk situations to coping with certain triggers. The imagined exposure and rehearsal can also be quite helpful to young people … They could lead the direction of treatment, set the goals, and identify problem behaviors. It allowed them to be in charge of their therapy.

### Stabilization

Seven codes were identified from 16 statements. Codes were: consolidation of previous skills (*n* = 4); useful layout and structure (*n* = 3); others noticed practicing skills (*n* = 3); adolescent tells others they are using their skills (*n* = 2); didn’t get the full benefit not getting to PC (*n* = 2); easy to apply (*n* = 1); and rehearsal for high risk helpful (*n* = 1). The theme is: *Easy application leading to positive adolescent change*. As one therapist reported, “there have been reports from family and unit staff that the young person put it into practice and used it, and sometimes a young person will tell them … This is what I’m doing and this is why I’m doing it, because this is what I’ve been taught.”

### Problem Solving/Avoiding High Risk

Six codes were identified from 10 statements. Codes were: understandable (*n* = 5); benefitted through worker consolidating skills (*n* = 1); no model conflict (*n* = 1); well structured (*n* = 1); materials provided were useful (*n* = 1); and skills to give adolescents (*n* = 1). The theme is: *Builds onto previous skills, and materials were clear to use*. Most therapists mentioned that FTM led to increased strategies and possibilities for young people, “You see them identify the problem and work through the steps. It lets them think about alternatives … when you start looking at actual behavior and avoiding the high risks, it makes sense to them.”

### Resolution of Trauma Memories

Seven codes were identified from 9 statements. Codes were: significant development in practice (*n* = 3); helped focus adolescents (*n* = 1); PC minimized rawness (*n* = 1); taught completely new technique (*n* = 1); like the idea we can process adolescent trauma memories (*n* = 1); didn’t get much practice (*n* = 1); and always consider PC as an option (*n* = 1). The theme is: *Gaining knowledge that trauma is treatable*. Two therapists described PC as helping them “minimize the rawness … before I wouldn’t have gone there, now I have it in the back of the mind, is this young person able to think about resolving trauma, and would progressive counting help.”

### Consolidation of Gains

Eight codes were identified from 13 statements. Codes were: repetition needed to deal with challenges (*n* = 2); clear rational for treatment and benefit for processing trauma (*n* = 2); opportunity for adolescent reflection (*n* = 1); adolescents developed new strategies (*n* = 1); useful framework to review and identify gaps (*n* = 1); future movies helpful in formulating a plan (*n* = 1); and material and activities helped adolescent understand how to deal with their experience (*n* = 1). Four comments, however, referred to not getting to consolidating gains and therefore did not fit with the theme of: *Discovering strategies to deal with past experience and for positive change*. One therapist’s comment capturing the value of the treatment framework to identify gaps for intervention was, “The framework sheet’s got all the aims of treatment. Even though I didn’t get to progressive counting, I would also recap and that helped to formulate a plan.”

### Anticipation of Future Challenges

Seven codes were identified from 15 statements. Codes were: learned to think about good and bad endings and make better choices (*n* = 4); made them think about future challenges (*n* = 3); developed motivation and understanding (*n* = 2); useful for intervention and future risk management (*n* = 2); and need the opportunity to practice on a regular basis and see the outcomes (*n* = 2). Two therapists’ comments did not fit the theme, i.e. ‘uncertain if it made a difference’ and ‘similar to relapse prevention.’ The theme is: *For most, opened up new ways of thinking and increased awareness of being able to make choices*. The therapist comment, “It made them think about what else was out there for them, what else could happen, like good and bad things, and thought right, this is what could happen and what could I do in that situation?” appears to reflect most therapists’ understanding of youth development of situational awareness and choice of strategy.

### Reported Care Staff Comments

Eight codes were identified from 19 statements. Codes were: managing anger (*n* = 4); talking more including past issues (*n* = 4); definitely beneficial (*n* = 4); care staff noticed changes (*n* = 2); the goal was put on the back of the room door (*n* = 2); adolescents wanted to work quicker, more intensively (*n* = 1); discussed future movies with staff (*n* = 1); and increased effort (*n* = 1). The theme is: *Care staff noticed visible changes in attitude and behavior*. For example, one therapist described, “It really made a change in the young person. One boy discussed his future movie within the unit. He’d set up his own goal, he had put his goal put on the back of his door from that session - he would replay it.”

### Benefit for Therapists

Twenty-two codes were identified from 69 statements. Codes were: helpful structured script (*n* = 12); increased awareness and understanding of trauma (*n* = 11); reduced planning time (*n* = 8); increased confidence in working with trauma (*n* = 8); discovered a way into addressing traumatization (*n* = 7); stimulated getting other trauma-specific training (*n* = 5); encouraged self-directed learning (*n* = 4); clear and structured pack (*n* = 3); helpful formulation and treatment plan (*n* = 3); an adolescent-led process (*n* = 2); consolidated psycho-therapy learning (*n* = 2); appreciated the opportunity to take part (*n* = 2); useful rational for treatment (*n* = 1); learned a lot for the future (*n* = 1); novel & beneficial (n = 1); can apply parts of FTM (*n* = 1); trained in treatment that processes trauma (*n* = 1); understandable structure for young people (*n* = 1); script useful with difficult adolescent responses/non-response (*n* = 1); looking beyond the behavior to what happened to adolescents (*n* = 1); teaching other staff about trauma (*n* = 1); and young people knew what was expected of you (*n* = 1). The theme is: *Increase in understanding and awareness of trauma exposure and recovery*. One therapist felt that the structure allowed for a great deal of flexibility despite being scripted, while another referred to increased discussion, reading, and understanding of trauma for unit staff.

FTM provided a structure that wasn’t too rigid. Even though it was scripted, because it was led by the young person as such, that is where you got your flexibility. You weren’t trying to force young people to do what they didn’t understand. As the therapist, initially it provided a lot of confidence in working with someone with trauma. Prior to that, I never felt confident with any of the other methods. Trauma has always been something I’d like to do something about it, but not sure what to choose. With this, I felt confident that we could work with young people and it would be successful. Discussion occurred about how this model developed and how processing might work … you started to think about it more, do more reading. I’ve started to think about it more in my psychological formulations, so talk about trauma more, think about trauma more … It’s been hugely beneficial in us helping the staff understand the presenting behaviors. I’ve got several examples, that are used with the staff and it helps them to look at it from a different perspective rather than just looking at the behavior.

### Therapist Skill Gains Trauma History Assessment

Nine codes were identified from 12 statements. Codes were: structured and trauma focused (*n* = 3); lack of previous experience (*n* = 2); think about presenting problems from a trauma lens (*n* = 2); understandable (*n* = 1); a new and different way of assessing (*n* = 2); ; led to clearer formulation (*n* = 1); know what was expected (*n* = 1); doable (*n* = 1); highlights impact of trauma more (*n* = 1); and now ask for more detail of events (*n* = 1). The theme is: *Overall increase in assessment accuracy and capacity*. Some therapists felt that they were more aware of trauma in assessment, as one stated, “I definitely think more about highlighting the impact of trauma within the assessments and in terms of presenting problems. Impulsivity and difficulty in managing emotions, thinking about the trauma in relation to those things.”

#### Trauma-Informed Case Formulation

Ten codes were identified from 14 statements. Codes were: gains in formulating treatment plan and delivering therapy (*n* = 4); shift from focusing on behavior to resolving the trauma (*n* = 2); built on current skills (*n* = 1), taught new skills (n = 1), more formalized approach (*n* = 1); useful quick formulation before intervention (*n* = 1); use a more complex formulation now (*n* = 1); needs to be set within the referral process (*n* = 1); and clear objectives and expectations (*n* = 1). One therapist reported they were inexperienced in delivering (*n* = 1). The theme is: *Delivery of therapy more efficient*. Some therapists highlighted that focusing on trauma aided efficient working, as one commented “Treatment contracting is different because we were generally taught, what’s the behavior that’s a difficulty, and how are we going to look at it? We now tag on, can we resolve the trauma. Before we would have just left the trauma.”

#### Trauma Within Report Writing

Eleven codes were identified from 22 statements. Codes were: definitely focus on trauma in reports now (*n* = 5); little changed as limited by standard report format (*n* = 3); helps explore best practice (*n* = 3); helped consolidate thinking and formulation (*n* = 2); trauma as an important factor in understanding and addressing children’s behavior (*n* = 2); didn’t focus much on the trauma (*n* = 2); no opportunity and no awareness of writing trauma into reports (*n* = 1); talk to parents about trauma in reports (*n* = 1); can speak about trauma more (*n* = 1); only write feedback to adolescents (*n* = 1); and reports need to change (*n* = 1);. The theme is: *Lack of experience in trauma report writing has been addressed for some*. One therapist, for example, emphasized the value of focusing on what had happened to a young person, and their resultant behavior, in reporting back to parents and staff. “Within my assessments and formulation, I touch more on trauma now. Which means I speak to parents, staff, and young people about it in terms of, this happened previously, this is how it impacts on your behaviors.”

#### How Speak to Parents, Staff and Other Agencies

Ten codes were identified from 21 statements. Codes were: more focused in trauma talk with staff (*n* = 5); tend to use CBT as more experienced (*n* = 4); include rational on understanding trauma and how to deal with it (*n* = 2); may need separate training on how to give parents skills to support adolescent during program (*n* = 2); need training on how to include care workers to look at child’s environment (*n* = 2); better informed (*n* = 2); more confidence (*n* = 1); examples of good practice needed for stakeholders (*n* = 1); no change (*n* = 1); not enough information for parents (n = 1); and not enough experience of FTM (*n* = 1); The theme is: *Greater knowledge, skill, and confidence in speaking about trauma to others, but a need for training in involving parents and care workers*. As well as increased confidence, enabling staff to be more aware of responses that may traumatize was highlighted by therapists. “It’s informed myself a lot, it’s given me more confidence to speak about it because I’m speaking from an informed standpoint rather than basic knowledge … supporting staff to think about behavior change and what they do, that might re-traumatize, and teach similar things that the young person’s been taught through the trauma. Much more focused on that now.”

#### Trauma-Sensitive Environment

Seven codes were identified from 12 statements. Codes were: consider strategies for self-soothing for adolescents (*n* = 3); FTM complimented focus on trauma sensitive environment (*n* = 2); and good preparation for a safe environment (*n* = 2). Negative codes were: not sure of influence (*n* = 2); already aware (*n* = 1); not much on wider trauma-sensitive environment (*n* = 1); and we don’t focus on the environment much (*n* = 1). The theme is: *Mixed responses revealed some staff are already trauma-aware*. Thinking about strategies young people use to calm and the complimentary nature of FTM in developing a safe environment was noted by a number of therapists, “I think about strategies young people can use to self soothe, to self-calm … the environment is all about trauma, and that was complemented by FTM, because of the ‘prepare your environment’ and the importance of making it a safe place for them.”

#### Trauma in Secure Care Transition Meetings

Nine codes were identified from 26 statements. Codes were: clear communication of understanding and processing trauma (*n* = 6); increase in trauma contributions (*n* = 5); not part of the transition process (*n* = 4); increased awareness following FTM training (*n* = 3); raised awareness of trauma and treatment for others (*n* = 2); lack of understanding of trauma in meetings (*n* = 2); provides other language to describe trauma (*n* = 2); improved confidence in meetings (*n* = 1); and preference to be involved (*n* = 1); The theme is: *Trauma now reported in transition meetings*. Therapists reported a growth in confidence and a more detailed approach to discussing trauma recovery, “It improved confidence going into meetings with trauma … and I did have a better understanding of how we would target the trauma giving them the details of the behavior, details of the program, and how you would process memories”.

### Barriers to FTM

Twelve codes were identified from 22 statements Codes were: not supported enough till emotionally ready (*n* = 3); complexity of adolescents difficulties and not responding (*n* = 3); heavy workloads (*n* = 2); adolescents’ low emotional intelligence (*n* = 2); under-resourced therapist teams (*n* = 2); too many staff and staff role changes (*n* = 2); different disciplines not fitting together (*n* = 2); paradigm clash (*n* = 2); too many adolescents (*n* = 1); lack of professional breadth (*n* = 1); short stays (*n* = 1); and over focus on assessment (*n* = 1); The theme is: *Lack of skills, time, and resources are a challenge*. Typical staff challenges were quoted as “Workloads and the other programs you’ve got to deliver. All the places are looking at what programs should look like now. The other thing is maybe short stays as young people are only in three weeks and you may not have the time. Normally the first three weeks are just an assessment period.”

### Facilitating FTM

Nine codes were identified from 16 statements. Codes were: can give longer than 50 min sessions (*n* = 4); information for the key worker to support FTM (*n* = 3); choice of therapist works (*n* = 2); not having other cases to work on (*n* = 1); intensive sessions to get it over quickly for adolescent (*n* = 2); flexibility (*n* = 1); support network (*n* = 1); reduced preparation time enables extra sessions (*n* = 1); and let the adolescent determine time, and priority of what to work on (n = 1); *The theme is: Need for flexibility and support to facilitate FTM*. The opportunity for longer more concentrated sessions was seen as helpful by most therapists, “I liked FTM because you could do longer as long as the young person was up for it. It didn’t have to be an hour … some benefitted from it concentrated … having that flexibility to let the young person determine what they think is the priority is helpful.”

## Discussion

The current study discovered that FTM can be applied in secure facilities as well as residential settings, perhaps because both types of facility cater to youth who have experienced neglect and cumulative abuse, and present with PTSD and other trauma symptoms (Barron and Mitchell 2017a, b). As with residential studies, therapists’ in secure facilities perceived a range of gains for youth who experienced FTM (Farkas et al. 2010). Similar to youth reports in Barron and Tracey’s (2017) cluster case study in a secure facility, youth were perceived by therapists in the current study to made gains in motivation, stabilization, problem-solving, anticipating risks, and imagining more hopeful futures (Greenwald et al. 2003). These perceived gains reflected program components, suggesting each phase of FTM may make a valuable contribution towards positive outcomes (Greenwald 2013). Significantly, gains observed by care staff, indicated that youths had applied FTM strategies in care and education settings. Increased effort, managing emotions, talking about past harms, and applying coping strategies were all reported. Previous juvenile justice studies highlight that such gains tend to be underpinned by youths’ understanding of trauma and its impact on behavior and health. Further benefits can also be accrued for family and community relationships (Ford and Blaustein 2013). Indications are then, wider social benefits may occur and that these should be included in future program evaluation.

Gains were not only perceived by therapists for youth but also for themselves, and their facilities. Adding to the gains of satisfaction and reduced reactivity found in previous residential qualitative studies (Greenwald et al. 2003, 2008), the current study found perceived increases in therapist trauma-informed knowledge, skills, and confidence, as well as the ability to talk about trauma with staff, parents, and agencies. In contrast to behavior risk and criminogenic models, FTM was perceived to support the growth of a trauma-sensitive environment (Barron and Tracey 2017). For some therapists, traumatization and resultant symptomology was increasingly discussed in placement transition meetings. In short, it would appear FTM enabled a shift for most therapists in understanding trauma as the underlying driver of behavior. Burrell, (2013) identified such an understanding as foundational to a trauma-informed approach for youth in juvenile detention. Despite the promise of FTM, there were a number of barriers to implementation in secure facilities. Paradigm clashes occurred where cognitive behavioral therapy (CBT) was the standard approach used. Experienced therapists’ capacity to tolerate feeling deskilled in implementing a new brief exposure therapy appeared to limit the extent of FTM delivery. Other therapists reported that insufficient priority and time was given to therapy within their facilities, and that the high volume of cases led to restrictions in the numbers of youth who could be seen. Further, most therapists were new to trauma-informed practice and found it difficult to apply FTM to a youth population with complex trauma histories and symptoms. Had therapists had the opportunity to treat youth with a range of severity of problems, this may have provided a more graded way of learning FTM. Therapists also perceived youths’ limited emotional awareness as one reason why youth in secure facilities needed more than expected preparatory work prior to the brief exposure component of FTM. Finally, many placements were of short duration of 1 to 3 months. This along with an institutional priority for assessment and reporting, rather than intervention, created competing demands on limited time which led to therapists under-practicing. The latter may have underpinned many of the above problems as therapists’ who have time protected for therapy do not appear to experience the same levels of challenge (Greenwald et al. 2003, 2008). Juvenile detention literature highlights further barriers to implementation for trauma-specific programs. Ford and Blaustein (2013) report that facility staff can hold the erroneous belief that punitive approaches work, and are less open to supportive models of intervention. A lack of training and supervision for staff in traumatization, and empathic responding can also lead to a higher risk of staff becoming dysregulated. These factors often result in an over focus on youth taking responsibility for their behavior and an over use of restraint, and seclusion (Ford and Blaustein 2013).

Counter to these challenges, therapists identified a range of facilitative factors for FTM in the secure setting. Enabling youth, who struggle to trust adults, to choose their therapist was reported as enhancing therapeutic alliance, a feature identified within the common factors research (Greenwald 2013). The capacity to deliver longer intensive sessions was seen by therapists as fitting well with youth who wanted to resolve their trauma in as short a time as possible. Information to care workers on how to support therapy and youths’ outcomes was perceived to be essential in fostering collaborative practice to enhance the efficacy of FTM. A reduced number of cases and time prioritized for therapy was seen as necessary to sustain trauma-informed practice over time. As a possible way forward, Burrell (2013) identified the reduction of administrative tasks as a way of enabling more time for therapy. Finally, as part of addressing these organizational issues, Barron and Mitchell (2017a, b), in a study that evaluated manager perspectives in secure facilities, highlighted the need for manager training in understanding youth traumatization and strategic planning in trauma-informed approaches.

## Limitations

The current study sought novice therapist perceptions of FTM. Perceptions of others, e.g. youth, parents, and agencies are likely to have led to the identification of further issues. Indeed, the sample size of ten therapists may not have been enough for the saturation of issues (Orne and Bell 2015). Therapists were novices in FTM and located in Scotland and as such, they may not represent perceptions of more experienced therapists and those working beyond Scottish borders. Although therapists reported that their program fidelity was high, therapists did not always complete the whole program, especially the progressive counting phase. Therapists were also hamstrung by not having adequate time to deliver what they were taught. Had therapists been able to practice more, responses to interview questions may have differed in a variety of ways. Therapists were predominantly female providing little evidence of male experience. As no attempt was made to measure therapist program fidelity, some therapists may have been commenting on adapted versions of FTM. Given therapist perceptions may not match actual youth behavior change, conclusions are exploratory and speculative (Smith 2015). Quasi-qualitative analysis can be criticized for being overly compartmentalized and the frequency of occurring statements may not reflect the importance of any individual statement in relation to therapeutic efficacy.

## Conclusions

The current study sought to investigate novice therapist perceptions of FTM in secure facilities in Scotland to identify the outcomes, and adaptations needed for future delivery, and evaluation. Therapists perceived FTM achieved benefits for youth, therapists, facilities, and agencies. Youth were perceived to make gains in motivation, awareness, and management of emotions, consequential thinking, anticipating risks, and identifying future goals. For therapists and facilities, FTM was perceived to be helpful in facilitating trauma-informed assessment, the creation of trauma-sensitive environments, treatment planning, and enhancing trauma-informed communication with care staff, parents, and agencies. In contrast, however, therapists were concerned about the complexity of youth difficulties, the lack of institutional prioritization for therapy, and the risks of not completing therapy because of short-duration placements. Such barriers risked undermining delivery of what may be a promising program for youth in secure facilities. In light of the lack of literature evaluating FTM in juvenile detention, the current study is relevant to both international research and practice contexts.

### Recommendations for Practice

As this was a small sample qualitative study, recommendations for practice are tentative. Indications are prioritizing time for FTM is important as is the opportunity for more intensive longer sessions. Overloading therapists with non-therapy responsibilities took the bulk of their time and is experienced by therapists as counterproductive. Psychoeducation for care staff, parents, and outside agencies who support youth in secure appears to facilitate program efficacy. Stability of staffing is an issue for delivering therapy consistently and effectively. Utilizing FTM as assessment as well as intervention may be an efficient way of working for facilities responding to short duration placements. Some youth in secure facilities may need work on awareness of emotions in the early phases of FTM. Implementation of FTM in Scottish secure accommodation needs to be set within the above recommendations. Finally, although findings from a recent cluster case study suggests FTM can reduce internal distress for youths (Barron and Tracey 2017), staff in the current study expressed the need for ongoing supervision to enable FTM to be delivered effectively.

### Recommendations for Research

Future qualitative research could explore and compare the perceptions of therapists, youth, care staff, managers, parents, and agencies in the experience, and efficacy of FTM. Therapists of differing gender and experience, with sufficient time allocated for therapy, need to be included. Further research is needed into facilitating factors and overcoming barriers of delivery of FTM in secure facilities. Above all, future research needs to test the gains perceived by therapists and others. More robust evaluative research needs to involve quasi-experimental, experimental, with mixed method designs. The latter enables participant experience to be assessed alongside therapy outcomes. Longitudinal studies would enable the assessment of whether gains were maintained over time.
